# The Identification of a New Gene *KRTAP 6-3* in *Capra hircus* and Its Potential for the Diameter Improvement of Cashmere Fibers

**DOI:** 10.3390/genes16060721

**Published:** 2025-06-19

**Authors:** Jian Cao, Zhanzhao Chen, Jianmin Zhang, Liang Cao, Shaobin Li

**Affiliations:** 1 College of Bioengineering, Jiuquan Vocational and Technical University, Jiuquan 735000, China; caojian@st.gsau.edu.cn (J.C.);; 2 Gansu Key Laboratory of Herbivorous Animal Biotechnology, Faculty of Animal Science and Technology, Gansu Agricultural University, Lanzhou 730070, China

**Keywords:** *Capra hircus*, *KRTAP6-3*, cashmere quality, mRNA expression localization

## Abstract

Background: Cashmere is one of the important economic products of goats, and the KRTAP gene family, as an important family of regulatory genes in the growth process of cashmere fiber, largely affects the quality of cashmere. Methods: In this study, the *KRTAP6-3* gene was identified and located on goat chromosome 1 using a goat genome homology search combined with a phylogenetic tree approach. The Longdong cashmere goat *KRTAP6-3* gene variation and its effect on cashmere quality were explored by using the polymerase chain reaction single-stranded conformation polymorphism (PCR-SSCP) technique, in situ hybridization, and the allele presence/absence model. Results: The results identified a total of six SNPs in *KRTAP6-3*, three of which were located in the coding region and two of which were synonymous mutations, in addition to 45- bp deletion sequences detected in alleles C and F. Moreover, the *KRTAP6-3* mRNA showed a strong expression signal in the cortical layer of the primary and secondary follicles in the inner root sheaths, as well as in the cells of the hair papillae and the matrices during the anagen phase, and signaling at the sites described above is attenuated during the telogen phase. The presence of allele C was associated with increased MFD (mean fiber diameter) (*p* < 0.01). The MFD of goats with allele C genotype (genotype AC) was significantly higher (*p* < 0.05) than that of goats without allele C genotype (genotypes AA and AB). Conclusions: This indicates that genetic variation in the *KRTAP6-3* gene in goats is significantly associated with cashmere traits and can serve as a candidate gene for molecular markers of cashmere traits.

## 1. Introduction

Cashmere, produced from the secondary hair follicles of the cashmere goat, is one of the finest natural fibers in the world [1]. The reason why cashmere is very precious is not only because of the scarcity of production, but, more importantly, because of its excellent quality and characteristics, it is considered to be ‘fiber gem’ or ‘fiber queen’. Its characteristics involve it being an all-textile raw material that human beings can use and are incomparable, and, therefore, it is also known as ‘soft gold’ [2]. As one of the most important sources of economic value for goats, differences in the traits of cashmere can have an impact on the economic benefits of cashmere [3] and are of increasing interest due to the fact that cashmere is a natural and sustainable raw material. Over the past two decades, China’s cashmere production has shown a trend of first rising and then falling. The low economic returns from cashmere production may be one of the main reasons for the decline in cashmere production. The economic efficiency of cashmere depends on its own quality. Under equal cost conditions, higher-quality cashmere often leads to higher incomes for farmers, so researchers expect to use modern biotechnology to actively and effectively improve the quality of cashmere. The composition of keratin-related proteins and the genetic diversity of genes in cashmere are closely related to the quality of cashmere fiber [4], which provides important ideas and a basis for the selection and breeding research of cashmere goats.

Keratin-associated proteins (KAPs) are important protein components of cashmere fibers that form the matrix of cashmere fibers and, together with keratin, constitute the basic structure of cashmere fibers. The KRTAP gene family, as genes encoding KAPs, plays an important role in maintaining normal wool growth. It has been shown that variations in the KRTAP gene family members, sheep *KRTAP20-1* [5] and *KRTAP28-1* [6] genes, were significantly associated with MFD, suggesting that different genes may significantly affect the same wool fiber trait. Shaobin Li et al. [7] found significant associations between variation in *KRTAP26-1* and wool fiber yield, mean fiber length (MFL), MFD, and other traits, which, in turn, suggests that variation in the same gene may also affect multiple wool traits simultaneously. This is not only seen in sheep, but also in goats. The seasonal growth of cashmere fibers is a typical feature of cashmere goats [8], where the cashmere fleece undergoes cyclic renewal, and its growth process roughly consists of three phases: the anagen phase, the catagen phase, and the telogen phase [9]. Hair follicles generally consist of primary hair follicles (PHFs) and secondary hair follicles (SHFs), and breeding for cashmere goats is often focused on increasing the number of SHFs [10]. Researchers have found that the fineness of cashmere is the main factor affecting the quality of cashmere [11], so the mechanism of regulating the fineness of cashmere has received more and more attention from researchers. The average GC content of all sheep KRTAPs identified to date is relatively high, the processing capacity of the DNA polymerase is reduced by the high GC content, and the high GC content also increases the slippage of the DNA polymerase, leading to a higher rate of gene sequence mutations [12]. The correlation between the occurrence of nucleotide insertions and deletions and substitutions is universal across all prokaryotic and eukaryotic genomes tested [13,14,15]. KAPs generally contain high levels of cysteine, or glycine and tyrosine [16,17]. Cysteine plays an important role in the synthesis of cashmere fibers. It is the first limiting amino acid and forms the disulfide bond between KAPs and KIFs that are cross-linked to each other. A disulfide bond is a chemical bond that connects two cysteines in different peptide chains or in the same peptide chain, but no cysteine is present in sheep KAP36-1 [18], suggesting that there may be other forms of cross-linking or interactions between HGT-KAPs and KIFs. The presence of tyrosine in KAPs may play a crucial role, and the benzene ring in the tyrosine side chain allows tyrosine residues in HGT-KAPs to interact with other tyrosine residues or other aromatic amino acids [19]. In addition, in HGT-KAPs, tyrosine residues are usually surrounded by glycine residues, and if the glycine is close to the tyrosine, then the tyrosine residue can have greater freedom to move its benzene ring into the appropriate spatial orientation, (i.e., conformational freedom), which facilitates amino acid interactions. Tyrosine also has a reactive hydroxyl group at the end of its side chain, which can act as a hydrogen donor and, therefore, may form a hydrogen bond with another aromatic amino acid at the center of the benzene ring [20], which would make the ring layer interactions stronger and may lead to the further strengthening of the wool fibers while giving a degree of flexibility [21].

In recent years, the research on cashmere goats has become increasingly scarce. However, with the gradual improvement of people’s living standards, cashmere market demand is growing rapidly [22,23]; but, cashmere production is low and the contradiction between the market and the demand has been formed, so the progress of the improvement of goat breeds need to be further strengthened. With technological innovation, i.e., the use of genetic breeding to improve the yield and quality of cashmere [24], as a sustainable production of raw materials, the cashmere industry will have a broad space for development.

## 2. Materials and Methods

All experiments were approved by Gansu Agricultural University (Approval No. 2006-398) and complied with the Guidelines for the Keeping and Use of Laboratory Animals developed by the Ministry of Science and Technology of the People’s Republic of China.

### 2.1. Animal Species and Sources

Animals were sourced from the Qingyang City Breeding Company in Gansu Province, and 356 Longdong cashmere goats of about one year of age were selected and marked on the ears to differentiate them from each other, and to ensure that the animals were in similar physical condition, healthy, and kept under the same conditions.

### 2.2. Collection of Cashmere and Blood Samples

In mid-April, cashmere samples were collected from the central region of the posterior margin of the left scapula of each goat, and the corresponding cashmere traits were determined. At the time of cashmere collection, a partial blood sample was collected from the ear of each goat on an FTA card (Whatman, Maidstone, UK). The blood samples were naturally air-dried at room temperature and stored in a sample chamber for the subsequent extraction and analysis of goat genomic DNA.

### 2.3. Collection of Skin Tissues

Six Longdong cashmere goat rams at about three years of age, kept under the same conditions, were selected for the collection of skin tissues in March and October, i.e., during the anagen and telogen phases of wool development. The collection method was as follows: fix the goat so that the left side of the body is upward, remove the downy hair on the left side of the posterior edge of the scapula so that the skin is exposed, sterilize the skin with iodine volts and alcohol, and anaesthetize it with a needle locally; then, use a scalpel and tweezers to obtain the skin tissues of the Longdong cashmere goat of about 2 cm^2^. Completely spread out the collected skin tissue, place it between two glass slides to fix it, and then store it in the in situ hybridization solution.

### 2.4. Identification of the KRTAP6-3 Gene

The coding region sequence of sheep *KRTAP6-3* gene (GenBank accession number: KT725833) was used as a template, and BLAST (version: 2.14.0) was applied to search for homology in the goat genome (NC_030808.1). The homologous sequences were identified as the target sequences by constructing phylogenetic tree analysis.

### 2.5. DNA Extraction and Amplification of Target Fragments

Genomic DNA was obtained using the method described by Zhou et al. [25]. The *KRTAP6-3* sequence of sheep (GenBank accession number: KT725833) was used as a template, and the BLAST function of GenBank was used to search for Caprine Genome Assembly NC_030808.1. The sequence with the highest homology to the *KRTAP6-3* sequence of sheep was assumed to be the *KRTAP6-3* sequence of Longdong cashmere goat, and the specific primers were designed using primer 5.0 to amplify the sequence of goat *KRTAP6-3* gene. The primer sequence information was F: 5′-TCTACCCGAGAACAACCTC-3′, R: 5′-CTTCCATGATGCAGCCTAAC-3′. The annealing temperature was 57 °C; these primers were synthesized by Sangon Biotech Co., Ltd. (Shanghai, China).

### 2.6. Characterization of Genetic Variation in the Goat KRTAP6-3 Gene

Amplification product variation was analyzed using polymerase chain reaction single-stranded conformational polymorphism (PCR-SSCP) technology with silver staining for color development and gene sequencing. For specific experimental procedures, refer to the research conducted by Hua Gong et al. [26]. The amplification products were first subjected to polyacrylamide gel electrophoresis and, at the end of electrophoresis, the polyacrylamide gel was stained to show the bands according to the method described by Byun et al. [27]. Based on the staining results, the sequencing method was selected by determining the type of genotype of the sample. Genotypes included both pure and heterozygous types, and were sequenced by means of the direct sequencing of PCR amplification products and cut-gel sequencing, respectively. Sequencing was performed in both directions by Shanghai Sangong Bioengineering Co. (Shanghai, China). The optimal conditions for the SSCP electrophoresis of primed PCR amplification products were 180 v, 25 °C, and 16 h, and the concentration of the gel was 12%.

### 2.7. In Situ Hybridization and RT-qPCR

Specific primers were designed according to the sequence of the genes submitted in GeneBank (KT725833), which were used to detect the relative expression level of goat *KRTAP6-3* gene in Longdong cashmere goats at different times. RT-qPCR primers and the internal reference information are shown in Table 1. The RT-qPCR reaction was carried out with β-actin as the internal reference gene using the Applied Biosystems QuantStudio^®^ 6 Flex qPCR instrument, and the relative expression amount of *KRTAP6-3* gene was calculated by using the 2^−ΔΔCt^ method [28]. The morphologically intact body side skin of Longdong cashmere goats was selected, embedded in dipping wax, and sectioned, and then unfolded in DEPC water and baked overnight at 37 °C for spare use. The *KRTAP6-3* gene RT-PCR product was cloned and sequenced and then used to make the template for digoxin-labelled probe after repeated purification and DNA template denaturation. The steps of Roche digoxin labeling kit instructions were used.

### 2.8. Data Collation and Analysis

The collated data were analyzed for association between individuals with genotype frequencies and allele frequencies greater than 5% and cashmere traits using General Linear Mixed-Effect Models (GLMMs) using SPSS V24.0 (IBM, Armonk, NY, USA) software. ANOVA results showed that both gender and sire had an effect (*p* < 0.05) on cashmere traits in the model, so gender was fitted as a fixed factor and sire was fitted as a random factor in the model.

## 3. Results

### 3.1. The Result of KRTAP6-3 Gene Identification

**Figure 1:**
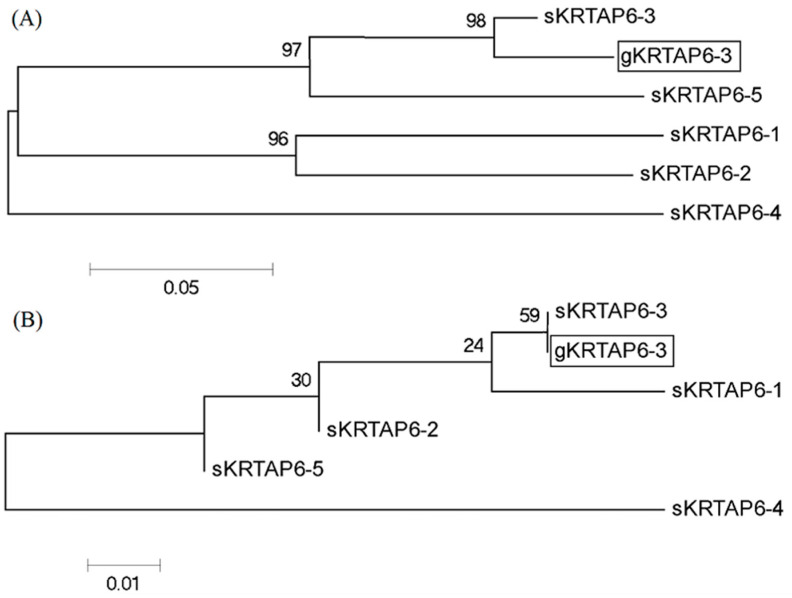
Phylogenetic trees constructed for the 300-bp sequences (**A**) upstream and (**B**) downstream of the coding region of the putative goat *KRTAP6-3* and sheep *KRTAP6-n*. The goat and sheep KRTAPs are indicated with a prefix “g” and “s”, respectively; the caprine *KRTAP6-3* sequences identified in the study are shown in a box, the same as below.

The coding region sequence of the sheep *KRTAP6-3* gene (GenBank accession number: KT725833) was used as a template, and the BLAST program was applied to conduct a homology search in the goat genome (NC_030808.1). The results showed that an open reading frame (ORF) with a size of 255-bp and 89% homology to the coding region sequence of the sheep *KRTAP6-3* gene was searched. The fragment is located on goat chromosome 1. Since sheep *KRTAP6-n* has high sequence similarity in the coding region, in order to confirm that this 255-bp ORF is the goat *KRTAP6-3* gene, the upstream and downstream 300-bp gene sequences of this ORF were further analyzed in this experiment, respectively, and, through the construction of the phylogenetic tree analysis, the amplified ORF was found to be the closest to the sheep *KRTAP6-3* gene sequence and more distant from other *KRTAP6-n* gene sequences as compared to other *KRTAP6-n* sequences in sheep. The upstream and downstream sequences had the closest genetic distance to the sheep *KRTAP6-3* gene sequence, whereas they were more distant from the other *KRTAP6-n* gene sequences, indicating that this sequence was the goat *KRTAP6-3* gene (Figure 1).

**Figure 2:**
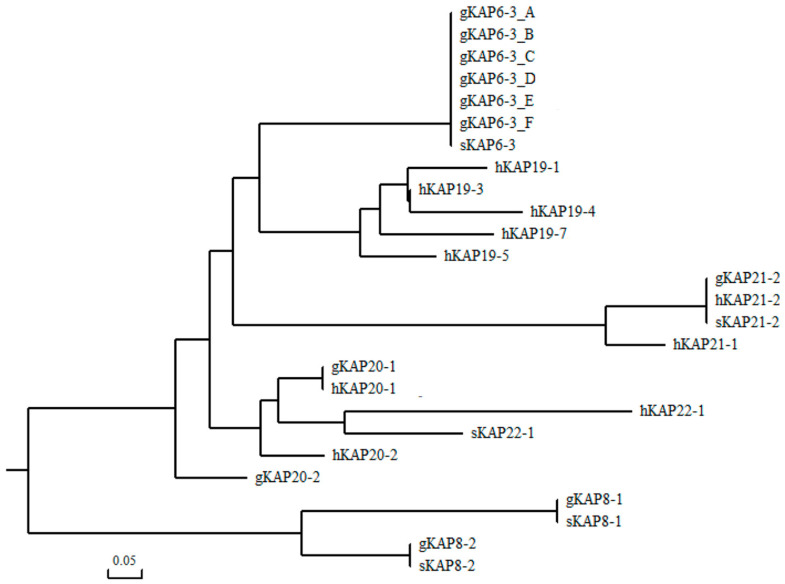
Phylogenetic tree constructed with newly discovered goat *KRTAP6-3* sequences and other HGT-KAPs found in goats, sheep, and humans.

The amino acid sequences of all HGT-KAPs already identified in humans, sheep, and goats were downloaded from the NCBI database, and phylogenetic trees were constructed with the six goat *KRTAP6-3* sequences obtained in this experiment. The results showed that the newly identified goat *KRTAP6-3* sequences clustered together with sheep *KRTAP6-3* with the closest genetic distance and clustered more distantly with other known HGT-KAP sequence genes (Figure 2). Combined with the results of 300-bp gene sequence analysis upstream and downstream of the newly identified ORF of the goat *KRTAP6-3* gene, this suggests that the six newly identified sequences are for the goat *KRTAP6-3* gene.

### 3.2. Genetic Characterization of the KRTAP6-3 Gene

**Figure 3:**
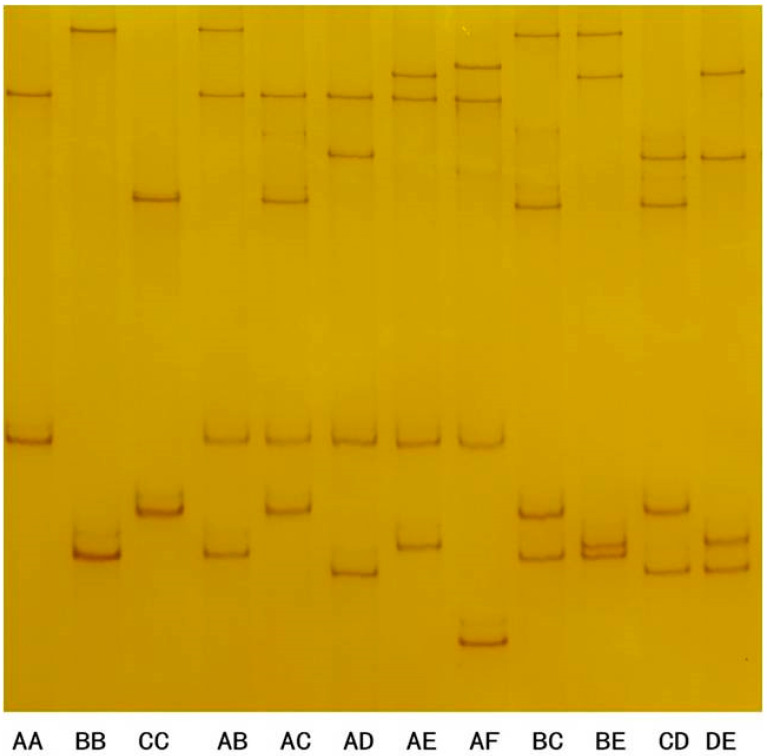
PCR-SSCP results of *KRTAP6-3* in Longdong cashmere goats.

After the polyacrylamide gel electrophoresis analysis of 337 Longdong cashmere goats, a total of 12 PCR-SSCP band types were identified (Figure 3), and six specific bands were identified after discrimination, which appeared individually or in combination in the tested individual goats.

The subsequent DNA sequencing of the PCR amplicons showed that the six SSCP bands represented six different nucleotide sequences, all of which differed from the DNA sequences of the goat genome (NC_030808.1) but showed 89% similarity, indicating that these six sequences represented the alleles of goat *KRTAP6-3*, and were named as alleles A, B, C, D, E and F, with allele frequencies A-F of 62.8%, 18.2%, 15.3%, 1.5%, 1.8% and 0.4%, respectively, and allele A being the advantageous allele. These six alleles in the form of pure and heterozygous individuals comprised a total of 12 genotypes AA, BB, CC, AB, AC, AD, AE, AF, BC, BE, CD, and DE with genotype frequencies of 39.8%, 2.7%, 2.7%, 24.3%, 17.8%, 0.9%, 2.1%, 0.9%, 6.2%, 0.6%, 1.2%, and 0.9%, respectively (Table 2). The AA genotype was the advantageous genotype.

**Figure 4:**
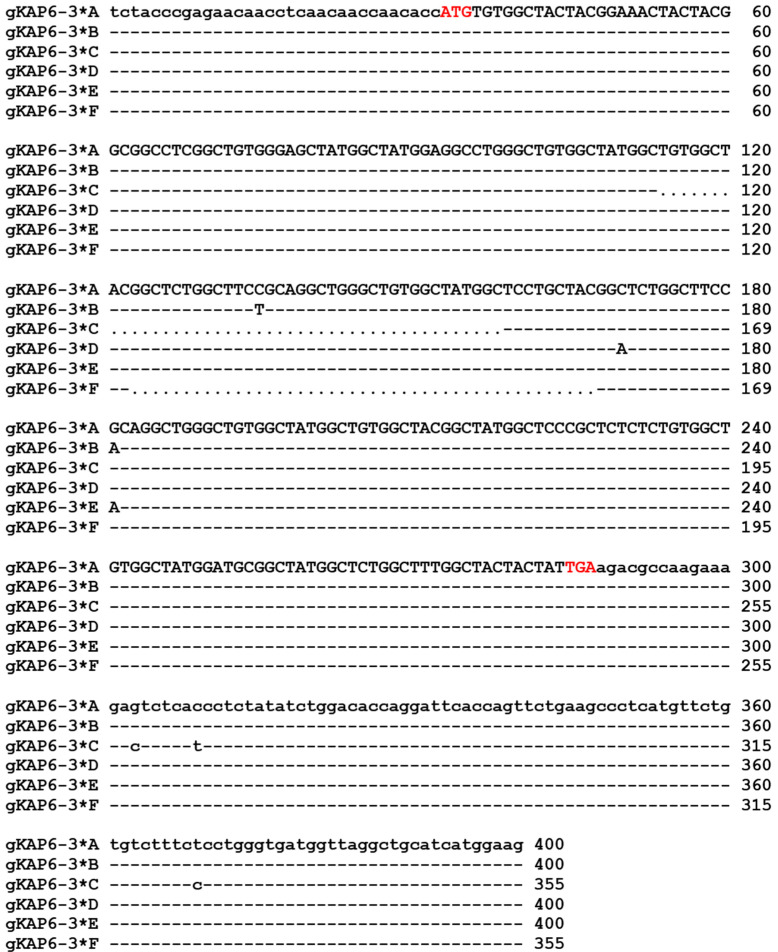
Sequence alignment of goat *KRTAP6-3*. Note: nucleotides in coding and non-coding regions are represented by upper- and lowercase letters, respectively. Dashes indicate nucleotide sequences that are identical to the sequence above, and nucleotide deletions are indicated by dots. Transcription start and stop codons are indicated by red letters.

**Figure 5:**
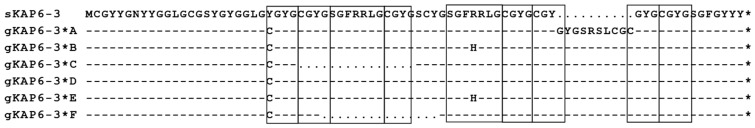
Alignment of the predicted amino acid sequences of the *KRTAP6-3* gene identified in goats and sheep. Dots represent amino acid deletions. Goats and sheep are labelled with the prefix “g” and the prefix “s”, respectively. Repeat sequences are indicated by boxes.

Sequencing results showed that six SNPs were identified in six nucleotide sequences (Figure 4), three of them were located in the coding region, c.103C/T and c.138C/A were synonymous mutations, c.149G/A was a non-synonymous mutation, which resulted in the change of arginine to histidine at position 50 (p.Arg50His), and the other three SNPs were located in the 3′UTR region, which were c.*16G/C, c.*22C/T, and c.*82T/C. In addition, sequences C and F have a deletion of 45 bp each (c.82_c.126delTGTGGCTACGGCTCTGGCTTCTGCAGGCTGGGCTGTGGCTATGGC and c.91_c.135delGGCTCTGGCTTCCGCAGGCTGGGCTGTGGCTATGGCTCCTGCTAC). The six allele sequences of goat *KRTAP6-3* encode polypeptide chains consisting of 84 or 69 amino acid residues (Figure 5). Among these three polypeptide chains, glycine and tyrosine had the highest content of 39.13–39.29 mol% and 21.19–23.19 mol%, respectively, cysteine had a moderate content (13.04–13.10 mol%), and other amino acids had a low content, such as serine 8.33–8.70 mol%, leucine 5.80–5.95 mol%, arginine 4.35–5.95 mol%, phenylalanine 2.90–3.57 mol%, methionine, and aspartic acid 1.19–1.45 mol%. Alleles C and F both have deletions of 15 amino acid residues (p.28_c.42delCGYGSGFRRLGCGYG and p.31_c.45delGSGFRRLGCGYGSCY) compared to alleles A, B, D, and E. In addition, four amino acid or seven amino acid sequence repeats (CGYG or SGFRRLG) were found in the amino acid sequence encoded by goat *KRTAP6-3*.

### 3.3. Localization and Relative Expression Levels of KRTAP6-3 mRNA at Different Periods of Time

**Figure 6:**
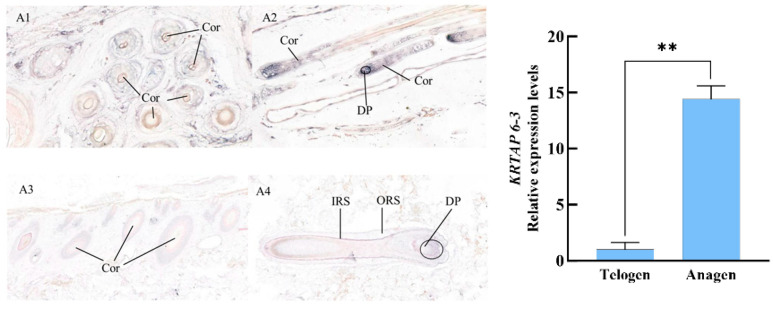
Expression localization and expression level of *KRTAP6-3* mRNA in the skin of Longdong goats at different periods of time. Note: **A1** and **A2** are anagen-phase-transverse and longitudinal cuts (30×), and **A3** and **A4** are telogen-phase-transverse and longitudinal cuts (30×), respectively. IRS, ORS, Cor, DP, and Mx are the inner root sheath, the outer root sheath, the cortical layer, the dermal papilla cells, and the hairy parent cells, respectively, same as below. **: Indicates the existence of extremely significant differences.

The results of the study on the relative expression levels of the newly discovered gene *KRTAP6-3* mRNA in different stages of goats showed that the gene was expressed in both the anagen and telogen, and the expression level was significantly higher in the anagen than in the telogen, with a multiplicity of difference of 14.42 times. A1, A2, A3, and A4 are the expression results of the *KRTAP6-3* mRNA of Longdong cashmere goats in anagen and telogen skin, respectively. The results showed that the hair follicle structure was more complete and clear and the cellular arrangement was more tightly ordered when the hair follicle was in the anagen phase compared to the telogen phase; the *KRTAP6-3* mRNA was strongly expressed in the cortical layer of the hair shaft of the primary and secondary hair follicles, the inner root sheath, and the hair papilla cells and hair matrices of Longdong cashmere goats during the anagen phase; and the expression of the signals in the above-described sites was weakened during the telogen phase (Figure 6).

### 3.4. Association Analysis of KRTAP6-3 Gene with Cashmere Traits

In the allele presence/absence model, among 337 Longdong cashmere goats, only three of the six alleles detected had frequencies greater than 5%, namely A (62.8%), B (18.2%), and C (15.3%). The other alleles, D, E, and F, had frequencies lower than 5% and were, therefore, excluded from the association analysis, leaving only the remaining 315 for statistical analysis. The results showed that the presence of allele C was associated with an increase in the mean diameter of cashmere (13.5 ± 0.04 μm in its absence; 13.9 ± 0.12 μm in its presence; *p* = 0.003), i.e., the presence of the allele C led to an increase in cashmere diameter. The remaining two traits measured were not associated with the presence (or absence) of alleles A, B, and C (*p* > 0.05) (Table 3).

Genotypes with less than 5% frequency were excluded from the association analysis with cashmere traits, including genotypes BB, CC, AD, AE, AF, BE, BC, CD, and DE. The association results showed that the mean fiber diameter was significantly higher in the AC genotype than in the AA and AB genotypes (*p* = 0.010), which suggests that allele C is associated with an increase in mean fiber diameter (Table 4).

## 4. Discussion

The keratin-associated protein gene family has been extensively studied, and it has been found that multiple members of this gene family have an impact on the economic traits of cashmere. A new gene, *KRTAP6-3*, was identified in goats in this study, adding a new member to the HGT-KAPs gene family, which is located on chromosome 1. In phylogenetic analysis, the goat *KRTAP6-3* gene was closest to the sheep *KRTAP6-3* gene in genetic distance and clustered together in the phylogenetic tree, suggesting that the gene is *KRTAP6-3* for goats. It is noteworthy that the goat KAP6-3 protein possesses a conserved “MCGYYGNY” and “GSGFGYYY” sequence at the amino-terminal and carboxy-terminal ends, respectively, which are unique to KAP6 proteins in mammals. Up to now, the same structure has been identified in the sheep *KRTAP6* family genes [29,30].

The sheep *KRTAP6-3* [31] gene showed abundant polymorphism in Merino sheep. Similarly, *KRTAP6-3* was found to be equally abundant in Longdong cashmere goats. A total of six SNPs were detected in the 399-bp fragments amplified in the Longdong cashmere goat population, and there was a 45-bp deletion sequence in both allele C and allele F with different deletion sequences. Sheep and goats are different genera in the same family, and although the *KRTAP6-3* gene is polymorphic in both, the sequence variants detected in both are different. The difference is that the SNPs identified in the sheep *KRTAP6-3* gene were all located in the coding region, and most of them (80%) resulted in amino acid changes, with non-synonymous mutations dominating. In contrast, in goat, three of the six SNPs were located in the coding region, only one was a nonsynonymous mutation, and the remaining three SNPs were located in the noncoding region, and the polymorphisms observed in both may be derived from different mechanisms. Similarly, a 45-bp insertion/deletion was detected in the central region of the coding region of both goat and sheep *KRTAP6-3* genes, and this new insertion/deletion in goat *KRTAP6-3* further supports the idea that sequence length variation is also a feature of the KRTAPs gene family [30]. To date, length variants are present in all *KAP6* genes except *KRTAP6-4*, but the number of insertions/deletions found in the KAP6 genes varies, with length variants detected in higher numbers in the *KRTAP6-1* and *KRTAP6-3* genes, 57-bp and 45-bp, respectively, and in lower numbers in the *KRTAP6-5* gene, 18-bp [29]. A 45-bp deletion was detected in goat *KRTAP6-3* alleles C and F. The effect of allele F on cashmere fiber traits was not analyzed because the genotype frequency of allele F was less than 5%, and only the effect of C on cashmere fiber traits was analyzed using the presence of deletion model, and a significant correlation was found between allele C and cashmere mean fiber diameter. The presence of allele C increases the diameter of cashmere fibers. The association analysis of the different genotypes of the *KRTAP6-3* gene with cashmere fiber traits showed that the average fiber diameter of the AB genotype, which did not contain allele C, was finer than the MFD ratio of the genotype AC, which contained allele C. There was no association of the different genotypes with cashmere yield and fiber curl length, which is consistent with the results of the presence/absence model. In the presence/absence model, the MFD of cashmere was smaller when allele C was absent than when C was present, suggesting that the deletion of allele C was associated with a reduction in MFD.

The results of this study show that there are significant differences in the localization of *KRTAP6-3* mRNA expression in telogen and anagen hair follicles, manifested as obvious differences in positive signal intensity and expression levels. During the anagen phase, strong mRNA expression signals were observed in the cortex and inner root sheath as well as papilla and hair matrix cells, while weaker signals were observed in the telogen phase, suggesting that the expression of this gene is spatio-temporally specific and exerts its main regulatory function mostly during the anagen phase. Furthermore, studies on *KRTAP6-3* in sheep have revealed that it also exhibits strong expression signals in the inner root sheath [32]; this indicates that the expression of the protein encoded by this gene is similar in goats and sheep. Compared with the telogen phase, the mRNA expression level of the gene was significantly higher in the anagen phase with a 14.42-fold difference, which was consistent with the results of previous studies [33]; expression levels of KRTAP gene family members were significantly differentiated between anagen and telogen phases. The newly identified gene had expression signals in the cortical layer, whereas no positive expression signals were detected in the medullary layer of the hair follicle, and it is hypothesized that the expression of this gene is more likely to benefit the improvement of cashmere traits. It has been shown that members of the KRTAP gene family show a typical co-evolutionary pattern, i.e., there is synergy between members of the same gene family, further confirming the similarity of expression patterns between members, and it has been suggested that the KRATP gene could play a more active role in improving the structure of cashmere fibers in domesticated mammals such as goats [34].

In the sheep *KRTAP6* family, both the 57- bp sequence deletion detected in allele C of *KRTAP6-1* [30] and the 45-bp sequence deletion detected in allele G of sheep *KRTAP6-3* [31] were significantly associated with wool fiber diameter. This suggests that sequence length variation may have an effect on cashmere wool fiber diameter. Sequence length variation in goat *KRTAP6-3* may affect cashmere fiber traits in several ways. First, the deletion sequences found in allele C and allele F may result in the deletion of 15 amino acid residues containing repetitive sequences in the central region of the protein, and these amino acid changes may affect fiber properties by affecting the structure, properties, or interactions with the IFs of the KAP6-3 protein. Secondly, deletion of the allele C amino acid sequence would result in an 18% reduction in the occurrence of the aromatic amino acids tyrosine (two deletions) and phenylalanine (one deletion) and a 50% reduction in the occurrence of the basic amino acid arginine (two deletions) in the protein. Aromatic and basic amino acids regulate the arrangement of intermediate filaments (IFs) by interacting with cation- π [35], and a reduction in the number of these residues may affect the strength of the interaction between KAP6-3 and keratin intermediate filament proteins (KIFs). Thirdly, 45-bp deletion also resulted in the absence of cysteine (two deletions) and glycine (six deletions). Cysteine is usually the first limiting amino acid for wool fiber synthesis and is required for the formation of disulfide bonds between KAPs and IFs, which play a very important role in wool growth. Although the exact role of glycine in HGT-KAPs has not been determined, the fact that glycine is the smallest amino acid and has no side chains may make HGT-KAPs proteins more flexible and, thus, better able to form tight structures with KIF proteins [31]. Finally, the deletion of the goat KAP6-3 protein also leads to a reduction in the number of residues that may be phosphorylated, which may result in changes in keratin solubility, the organization of keratin filaments, and interactions with other proteins [36,37], thus affecting the traits of cashmere fibers. The effect of the *KRTAP6-3* gene on cashmere fiber traits may also be caused by the independent action of the *KRTAP6-3* gene or by a cascade effect with upstream and downstream neighboring genes. However, as far as the association between the goat *KRTAP6-3* gene and the sheep *KRTAP6* subfamily of genes with MFD has been identified, it has the potential to be used as a molecular marker for cashmere fiber traits in goats. In subsequent studies, the association between genes and cashmere traits can be further verified through methods such as gene editing validation and cell experiments. The functional conservation of these genes in hair follicle development can be analyzed across different species. Based on modern genotyping technology, this research can be applied to molecular-assisted breeding in goats.

## 5. Conclusions

KAPs are the main protein components of goat cashmere and play a key role in the economic characteristics of cashmere. The newly identified gene *KRTAP6-3* in Longdong cashmere goats is significantly associated with MFD. Therefore, the newly discovered gene can be used as a candidate gene for molecular markers of cashmere traits for the improvement of the cashmere quality of breeds.

## Figures and Tables

**Table 1:** RT-qPCR primer information and amplification condition.

Gene	Primer Sequences	Length of Products (bp)	Annealing Temperature (°C)
*KRTAP6-3*	F: 5′-AAGCTATGGCTATGGAGGC-3′	99	60
	R: 5′-CCAGAGCCGTAGCAGGAG-3′		
β-actin	F: 5′-ACTACAGCAACTACTACGGTG-3′	112	60
	R: 5′-GGACAGCACCGTGTTGGCGTAGA-3′		

**Table 2:** Genotypes and their corresponding gene frequencies.

Genotype (*n*)	Genotype Frequency (%)	Genotype (*n*)	Genotype Frequency (%)
AA (134)	39.8%	AE (7)	2.1%
AB (82)	24.3%	CD (4)	1.2%
AC (60)	17.8%	AF (3)	0.9%
BC (21)	6.2%	AD (3)	0.9%
BB (9)	2.7%	DE (3)	0.9%
CC (9)	2.7%	BE (2)	0.6%

**Table 3:** Association between alleles in *KRTAP6-3* and variation in cashmere traits in Longdong cashmere goats.

Traits	Variant	Absent		Present		*p* Value
		Mean ± SE	*n*	Mean ± SE	*n*	
Cashmere yield (g)	A	415 ± 8.5	39	413 ± 5.1	276	0.805
	B	419 ± 4.5	203	406 ± 8.3	112	0.252
	C	412 ± 4.7	225	428 ± 13.1	90	0.313
Mean fiber diameter (μm)	A	13.5 ± 0.06	39	13.5 ± 0.05	276	0.629
	B	13.6 ± 0.04	203	13.5 ± 0.08	112	0.219
	**C**	**13.5 ± 0.04**	**225**	**13.9 ± 0.12**	**90**	**0.003**
Crimped fiber length (μm)	A	4.3 ± 0.10	39	4.2 ± 0.05	276	0.864
	B	4.2 ± 0.05	203	4.3 ± 0.09	112	0.556
	C	4.3 ± 0.05	225	4.2 ± 0.14	90	0.528

Note: Estimated marginal means and standard errors derived from general linear mixed-effects models that included “gender” as a fixed factor and “sire” as a random factor, respectively. *p* < 0.01 and *p* < 0.05 are shown in bold, same as below.

**Table 4:** Association analysis between *KRTAP6-3* genotype and cashmere traits in Longdong cashmere goats.

Traits	Mean ± SE			*p* Value
	AA (*n* = 134)	AB (*n* = 82)	AC (*n* = 60)	
Cashmere yield (g)	416 ± 8.6	407 ± 8.7	423 ± 15.1	0.314
Mean fiber diameter (μm)	**13.4 ± 0.08 ^b^**	**13.6 ± 0.08 ^b^**	**14.0 ± 0.14 ^a^**	**0.010**
Crimped fiber length (μm)	4.2 ± 0.09	4.3 ± 0.09	4.2 ± 0.16	1.000

a,b: The same letters indicate no significant difference, while different letters indicate significant difference.

## Data Availability

The authors affirm that all data necessary for confirming the conclusions of the article are present within the article, figures, and tables.
